# Editorial: Silk-Based Functional Biomaterials

**DOI:** 10.3389/fbioe.2021.721761

**Published:** 2021-11-19

**Authors:** Chengchen Guo, Shengjie Ling, Chunmei Li, Antonella Motta, Joaquim Miguel Oliveira

**Affiliations:** ^1^School of Engineering, Westlake University, Hangzhou, China; ^2^School of Physical Science and Technology, ShanghaiTech University, Shanghai, China; ^3^Department of Biomedical Engineering, Tufts University, Boston, MA, United States; ^4^Department of Industrial Engineering, BIOtech Research Center, University of Trento, Trento, Italy; ^5^Trento Unit, European Institute of Excellence on Tissue Engineering and Regenerative Medicine, Trento, Italy; ^6^3B's Research Group, I3Bs—Research Institute on Biomaterials, Biodegradables and Biomimetics, University of Minho, Headquarters of the European Institute of Excellence on Tissue Engineering and Regenerative Medicine, Barco/Guimarães, Portugal; ^7^ICVS/3B's—PT Government Associated Laboratory, Braga/Guimarães, Portugal

**Keywords:** silk, biomaterials, biomedical applications, degradation, tissue regeneration

Silk is a natural protein-based biomaterial produced by arthropods, including silkworms and spiders, that has been used for textiles historically due to its appealing physical properties such as good mechanical properties. Recent research effort has demonstrated that silk (e.g., silkworm silk) also possesses excellent biocompatibility and biodegradability, which significantly extends the use of silk to a much broader field, including tissue engineering, regenerative medicine, optics, and electronics. Compared to most synthetic materials, silk shows remarkable benefits toward some applications, particularly biomedical applications owing to the good combination of mechanical robustness, biocompatibility, and biodegradability. This research article collection focuses on some of the latest high-quality research with an emphasis on the development and applications of silk-based functional biomaterials.

A fundamental understanding of silk-cell interactions is critical for developing silk-based functional biomaterials. Kochhar et al. reviewed the biophysical influence of silk biomaterials on directing cellular behaviors. In the review, they explored how the physical and chemical properties of silks, both mulberry and non-mulberry based, affect cell behaviors including cell adhesion, cell proliferation, cell migration, and cell differentiation. Specifically, the influence of materials' compositions, mechanical properties, topography, and 3D geometry on regulating cell behaviors was discussed.

As a protein-based natural polymeric materials, silk present good biodegradability, particularly enzymatic biodegradability. To gain fundamental understanding of the enzymatic degradation kinetics of silk sponges, a common materials format used in tissue engineering and regenerative medicine. Jameson et al. developed a kinetic model that examines the effect of initial scaffold conditions on the rate of enzymatic degradation. In their study, two types of enzymes, including proteinase K and protease XIV were investigated. In addition, they held the silk concentration, polymer chain length and scaffold pore size constant while varying the crystallinity of the silk sponges and enzyme concentration. The experimental data was fitted with a modified Michaelis-Menten based kinetic model. The study provides a method to predict enzymatic degradation kinetics of silk sponge.

For silk-based implantable devices and tissue scaffolds, Kim et al. developed a dual-layer PEG-silk composite hydrogel mimicking the layered structure of articular cartilage. Natural silk fibers were used to construct the 3D silk fiber construct that was embedded in dual-layer PEG hydrogel. The composite construct had a compressive modulus of 744 kPa and facilitated the chondrogenic differentiation of human mesenchymal stem cells encapsulated in the composite. In addition, Yu et al. fabricated a biomimetic scaffold using silk nanofibers and hyaluronic acid (HA) for tissue repair. The silk-HA scaffold showed good biocompatibility and supports the cellular proliferation of bone marrow mesenchymal stem cells.

Tanaka et al. developed an elastin-silk fibroin double raschel knitted vascular graft with 1.5 mm in diameter. Water-soluble elastin was prepared from insoluble elastin by hydrolysis with oxalic acid. Compared to silk fibroin, elastin was less likely to adhere to platelets, while vascular endothelial cells were three times more likely to adhere. Silk fibroin-based artificial blood vessels densely packed with porous elastin were fabricated, and these prevented the leakage of blood from the graft during implantation, while the migration of cells after implantation was promoted. The elastin-silk grafts were implanted into the abdominal aorta of rats to evaluate their patency and remodeling ability. The results showed that there is limited adverse reactions, such as bleeding at the time of implantation or disconnection of the sutured end. In addition, vascular endothelial cells were present on the graft's luminal surface after 2-week implantation.

Silk can also be used as a good coating material to improve the biological performance of implantable metals or alloys. Zhou et al. performed a study on silk-coated Ti-6Al-4V alloy and found that the coated silk layer induced biomineralization at the alloy interface when incubated in simulated body fluid. Furthermore, the composite layer composed of a mixture of hydroxyapatite (HAP) and silk protein enhanced the osseointegration significantly. This study demonstrated a feasible way to use silk coating for enhancing the osteogenic property of the implants.

In summary, this article collection covers a broad range of topics from fundamental properties of silk-based materials to engineering silk-based materials or devices for biomedical applications. All the studies covered in the collection would initiate new ideas or direction for future development of silk-based functional materials.

## Author Contributions

All authors listed have made a substantial, direct and intellectual contribution to the work, and approved it for publication.

## Conflict of Interest

The authors declare that the research was conducted in the absence of any commercial or financial relationships that could be construed as a potential conflict of interest.

## Publisher's Note

All claims expressed in this article are solely those of the authors and do not necessarily represent those of their affiliated organizations, or those of the publisher, the editors and the reviewers. Any product that may be evaluated in this article, or claim that may be made by its manufacturer, is not guaranteed or endorsed by the publisher.

